# “HE JUST WANTS HIS SERVICES BACK”: IMPACTS OF HOME CARE WORKER DISRUPTIONS ON PATIENTS AND CAREGIVERS DURING COVID-19

**DOI:** 10.1093/geroni/igac059.2347

**Published:** 2022-12-20

**Authors:** Emily Xu, Patricia Kim, Meng Zhang, Jennifer Reckrey, Katherine Ornstein, Emily Franzosa

**Affiliations:** Icahn School of Medicine at Mount Sinai, New York, New York, United States; Icahn School of Medicine at Mount Sinai, New York, New York, United States; Icahn School of Medicine at Mount Sinai, New York, New York, United States; Icahn School of Medicine at Mount Sinai, New York, New York, United States; Johns Hopkins University, Baltimore, Maryland, United States; Icahn School of Medicine at Mount Sinai, New York, New York, United States

## Abstract

Home care workers (HCWs) have played a critical role in keeping homebound older adults safely at home during COVID-19, yet their essential work is often understudied. This study characterized the roles of HCWs during COVID-19 and examined how HCW service disruptions impacted patients and their caregivers. We performed a thematic analysis of medical records from 53 patients with HCWs in a home-based primary care practice in New York City. We abstracted unstructured clinical notes into a priori and emergent categories and identified core themes via team discussion. The following themes emerged: 1) Shifts to remote medical care and changing patient needs led to task shifting and new tasks for HCWs (i.e. getting food for patients experiencing food insecurity), 2) The risks associated with HCW tasks, such as exposure from caring for patients with COVID-19, increased during the pandemic, 3) Patient and family refusal of HCW services to avoid COVID-19 exposure as well as abrupt loss of HCW services due to HCW precarity or COVID-19 exposure left family caregivers with additional caregiving responsibilities, 4) Regulations surrounding return to work following COVID-19 exposure created additional difficulties in reinstating HCWs and left patients without adequate care, putting them at risk of hospitalization. In conclusion, pandemic-related disruptions created barriers to adequate home care, putting both patients and caregivers at risk. This analysis suggests a need for more robust HCW training and established regulations to protect HCW safety as well as a need for policies to support caregivers and ensure continuity of care during emergencies.

